# Dynamics of Sequestered Cryptophyte Nuclei in *Mesodinium rubrum* during Starvation and Refeeding

**DOI:** 10.3389/fmicb.2017.00423

**Published:** 2017-03-21

**Authors:** Miran Kim, Kirstine Drumm, Niels Daugbjerg, Per J. Hansen

**Affiliations:** ^1^Marine Biological Section, Department of Biology, University of CopenhagenHelsingør, Denmark; ^2^Marine Biological Section, Department of Biology, University of CopenhagenCopenhagen, Denmark

**Keywords:** nucleus enlargement, photosynthesis, sequestered chloroplasts, sequestered nucleus, *Teleaulax amphioxeia*

## Abstract

The marine mixotrophic ciliate *Mesodinium rubrum* is known to acquire chloroplasts, mitochondria, nucleomorphs, and nucleus from its cryptophyte prey, particularly from species in the genera, *Geminigera* and *Teleaulax*. The sequestered prey nucleus and chloroplasts are considered to support photosynthesis of *M. rubrum*. In addition, recent studies have shown enlargement of the retained prey nucleus in starved *M. rubrum* and have inferred that enlargement results from the fusion of ingested prey nuclei. Thus far, however, little is known about the mechanism underlying the enlargement of the prey nucleus in *M. rubrum*. Here, we conducted starvation and refeeding studies to monitor the fate of prey nuclei acquired by *M. rubrum* when feeding on *Teleaulax amphioxeia* and to explore the influence of the retained prey nucleus on photosynthesis of *M. rubrum*. Results indicate that enlargement of the prey nucleus does not result from fusion of nuclei. Furthermore, the enlarged prey nucleus does not appear to divide during cell division of *M. rubrum*. The presence of a prey nucleus significantly affected photosynthetic performance of *M. rubrum*, while the number of retained chloroplasts had little influence on rate of carbon fixation. We interpret results within the context of a model that considers the dynamics of ingested prey nuclei during division of *M. rubrum*.

## Introduction

*Mesodinium rubrum* (=*Myrionecta rubra*) is a common ciliate in coastal waters worldwide, where it sometimes causes red tides (Taylor et al., 1971; Lindholm, 1985). It is an obligate mixotroph, requiring both light and prey uptake for sustained growth and survival (Gustafson et al., 2000; Yih et al., 2004; Johnson and Stoecker, 2005; Hansen and Fenchel, 2006). Growth of *M. rubrum* is to a large extent phototrophic and closely linked to irradiance (Johnson and Stoecker, 2005; Johnson et al., 2006; Smith and Hansen, 2007; Moeller et al., 2011). Under culture conditions, *M. rubrum* feeds specifically on cryptophytes belonging to the genera *Geminigera* and *Teleaulax* (Hansen and Fenchel, 2006; Park et al., 2007; Myung et al., 2011; Hansen et al., 2012; Raho et al., 2014).

It has long been known that *Mesodinium rubrum* contains chloroplasts of cryptophyte origin (Taylor et al., 1971; Hibberd, 1977). Recent studies have shown that these chloroplasts are genetically similar to the prey on which *M. rubrum* is fed, indicating that chloroplasts are sequestered from the prey (Johnson et al., 2006, 2007; Hansen et al., 2012; Myung et al., 2013). Furthermore, cross-feeding experiments in which prey were switched from one species to another led to the replacement of chloroplasts in *M. rubrum*, depending upon the available prey species (Hansen et al., 2012; Raho et al., 2014). In starved *M. rubrum*, sequestered prey chloroplasts have been shown to divide along with cell divisions of the ciliate (Johnson et al., 2006; Hansen and Fenchel, 2006; Kim et al., 2016). Nevertheless, *M. rubrum* requires continuous acquisition of new chloroplasts and other cell organelles acquired through feeding for sustained growth.

Early works found that *M. rubrum* contains a single enlarged nucleus of cryptophyte origin, along with cryptophyte chloroplasts, other cell organelles, and cytoplasm (Taylor et al., 1969, 1971; Hibberd, 1977; Oakley and Taylor, 1978). This condition was believed to represent an incomplete endosymbiont, and the enlarged cryptophyte nucleus was referred to as a ‘symbiont nucleus’ (Hibberd, 1977; Oakley and Taylor, 1978). Later, however, it was verified that the symbiont nucleus, like the cryptophyte chloroplasts, is acquired by *M. rubrum* after feeding on prey (Gustafson et al., 2000; Johnson et al., 2007; Johnson, 2011; Hansen et al., 2012; Kim et al., 2016) and was termed ‘kleptokaryon’ by Johnson et al. (2007). For simplicity, the ‘enlarged acquired prey nucleus’ will be called the ‘symbiont nucleus’ in the remainder of the introduction.

The single symbiont nucleus observed in well-fed *M. rubrum* cultures (Johnson et al., 2007) is eventually lost following cell divisions of the ciliate subjected to prolonged starvation (Johnson and Stoecker, 2005; Johnson et al., 2007; Kim et al., 2016). However, the symbiont nucleus was still observed in all cells after the first cell division in prey starved experiments, indicating that the acquired prey nucleus divide at least one time inside *M. rubrum* (Hansen and Fenchel, 2006). Gustafson et al. (2000), as well as recent papers by Kim et al. (2016) and Nam et al. (2016) showed that well-fed *M. rubrum* retained additional prey nuclei which were smaller than the symbiont nucleus and of size similar to the nucleus of the prey species. Also, it has been observed that smaller prey nuclei become enlarged over time during prey starvation in *M. rubrum* (Johnson et al., 2007; Kim et al., 2016; Nam et al., 2016). The interpretation made by the authors of these recent studies was that acquired prey nuclei fused to make the symbiont nucleus. However, this interpretation was not experimentally tested and the exact mechanism underlying enlargement of the acquired cryptophyte nuclei in *M. rubrum* remains unknown.

In the present study, the fate of prey nuclei sequestered by *M. rubrum* was monitored during prey starvation and refeeding experiments using confocal microscopy. Changes in size and position of prey nuclei inside the ciliate were determined, and evidence of nuclear division or fusion was noted. Furthermore, the relationship between the presence of a retained prey nucleus and the photosynthetic efficiency and growth of *M. rubrum* was studied.

## Materials and Methods

### Cultures

Cultures of *Mesodinium rubrum* (MBL-DK2009) and *Teleaulax amphioxeia* (SCCAP K-0434; SCCAP) were established using single cells isolated from Helsingør harbor, Denmark, in 2009. Both species were grown in f/2 medium based on autoclaved seawater (Guillard, 1975) with a salinity of 30 and maintained in 24-well tissue culture plates (TPP, Switzerland). Both species were grown at 15°C ± 1.0 in a temperature regulated room, under a photon irradiance of 100 μmol photons m^-2^ s^-1^ (PAR, 400–700 nm), and on a light:dark cycle of 14:10. Light was provided by cool white fluorescent tubes (OSRAM, 58W, 840). Irradiance was measured (in seawater) at the level of incubation flasks using a light meter equipped with a spherical quantum sensor (ULM and US-SQS/L, Walz GmbH, Germany). *T. amphioxeia* was supplied as prey at a predator:prey ratio of approximately 10:1 when *M. rubrum* was transferred weekly to new medium.

### Experiment 1: Starvation of Well-Fed *Mesodinium rubrum*

**FIGURE 1:**
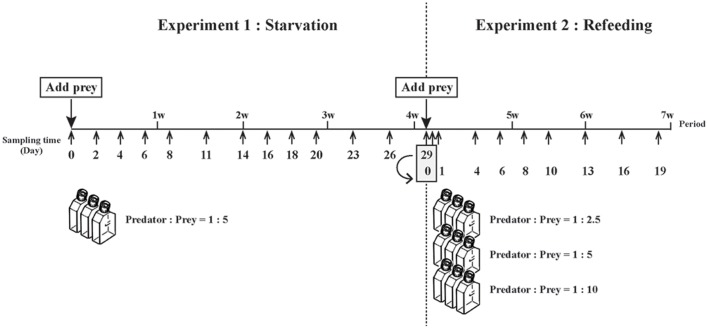
**Schematic illustration of experimental design used for starvation and refeeding experiments, respectively.** The two experiments were conducted over 7 weeks. Experiment 1, starvation of well-fed *Mesodinium rubrum*, was carried out for 4 weeks, with the starved cells at the end of that experiment immediately use in Experiment 2, refeeding of starved *M. rubrum*.

The first experiment was designed to monitor the change in photosynthetic performance and the number, size and position of prey nuclei during starvation of *M. rubrum. M. rubrum* cells were kept well-fed for 2 weeks by adding sufficient prey every 3 days to a culture grown in a 750-ml tissue culture flask (TPP, Switzerland). After 2 weeks of frequent feeding, the *M. rubrum* culture was allowed to deplete the prey, and absence of prey in the culture was confirmed under an inverted microscope at x40 magnification (Olympus CK2, Japan). A portion of the well-fed, but prey-free, *M. rubrum* and stock culture of *T. amphioxeia* were then added to a 750 ml tissue culture flask to achieve an initial predator:prey ratio to 1:5 and a *M. rubrum* cell concentration of ∼200 ml^-1^. From this stock culture, a 150-ml aliquot was transferred to each of three 270-ml tissue culture flasks. The flasks were placed on a shelf with light coming from the side at an irradiance of 60 μmol photons m^-2^s^-1^. Position of the flasks was changed sequentially once a day to minimize difference in light exposure between the flasks. Subsamples for cell enumeration and assessment of photosynthetic performance were withdrawn from each flask on 13 occasions during the experiment: on Day 0, 2, 4, 6, 8, 11, 14, 16, 18, 20, 23, 26, and 29 (**Figure 1**). For confocal microscopy, a single Day 0 sample was taken prior to distributing stock culture to replicate flasks, with samples taken from replicate flasks on all other days. pH was monitored at each sampling occasion directly in the flasks with a SenTix^®^41 pH electrode (WTW, Germany) connected to a pH meter (WTW, pH 3210, Germany), and calibrated with pH 7 and pH 10 standard buffers (WTW, Technischer, NIST, buffers). To avoid physiological effects of elevated pH in laboratory cultures (Hansen, 2002), half the volume of each experimental cultures of *M. rubrum* was removed and replaced with fresh f/2 medium on Day 8, 11, 14, and 20, when pH values were approaching to 8.5.

#### Cell Abundance and Growth Rate

Aliquots (2.3 ml) withdrawn from each flask were fixed with acid Lugol’s solution (final concentration 1%). Abundance of *M. rubrum* and *T. amphioxeia* was enumerated using a Sedgewick-Rafter chamber under the inverted microscope (Olympus CK40) at 100X and 200X. At least 400 cells were enumerated. Growth rates were calculated during the exponential portion of the growth phase using the following exponential growth equation:

$$\mu =InN_{1}-InN_{0}/t_{1}-t_{0}$$

where *N*_1_ and *N*_0_ are cell concentrations at time *t*_1_ and time 0, respectively, and *t*_1_-*t*_0_ is the time interval between samplings.

#### Measurement of Photosynthetic Activity (^14^C)

Two 2-ml aliquots were withdrawn from each flask, transferred to each of two 20-ml glass scintillation vials, and used for measurements of photosynthesis. Twenty microliter of NaH^14^ CO_3_ stock solution (specific activity = 100 μCi ml^-1^; Carbon-14 Centralen, Denmark) was added to each vial. One vial of each pair was incubated for 3 h in the same place as the experimental flask, and the other vial was kept in complete darkness by wrapping in aluminum foil. After incubation, a 100 μl sub-sample was withdrawn from each vial and added to a new vial containing 200 μl phenylethylamine for measurements of specific activity (Skovgaard et al., 2000). The remaining 1.9 ml were acidified with 2 ml 10% glacial acetic acid in methanol, and evaporated overnight at 60°C to remove all inorganic carbon. The residue in the vial was re-dissolved in 2 ml Milli-Q water before adding 10 ml of scintillation cocktail (Insta-Gel Plus, Packard, USA). All vials were vigorously shaken and then analyzed using a liquid scintillation counter (Tri-Carb 2910 TR, Perkin-Elmer). Photosynthetic activity (PA, pg C cell^-1^ h^-1^) was calculated as follows:

PA=DPM×[DIC]/C14×h×N

where DPM is disintegrations min^-1^ (in 1.9 ml) in the light corrected for dark value, DIC is the concentration of dissolved inorganic carbon (pg C ml^-1^), ^14^*C* is the specific activity (disintegrations min^-1^ ml^-1^), *h* is the incubation time, and *N* is the number of cells in the vial (1.9 ml). DIC concentrations were measured within a few hours using a total organic carbon analyzer (TOC-L, Shimadzu, Japan).

#### Confocal Microscopy

Location, number, and changes in size of prey nuclei inside *M. rubrum* were studied by confocal microscopy. Nuclei were stained using the fluorescent nuclear stain Hoechst 33258 (Invitrogen, USA) and plasma membrane stain using CellMask Green (Life technologies, Carlsbad, CA, USA). Subsamples (5–10 ml) taken from each flask were fixed in 1% glutaraldehyde (EMD Millipore, USA) at 4°C for 1 h. Fixed samples were stained with a combination of 25 μg ml^-1^ Hoechst 33258 and 0.25 X CellMask for 15 min in a dark chamber, then filtered through a 0.2 μm black polycarbonate membrane filter (Frisenette, Denmark), and finally washed with fresh autoclaved seawater to remove excess dye. A drop of immersion oil placed on both sides of a membrane filter was used to attach the filter to the microscope slide and coverglass. Nuclear size was measured directly from images taken at 600X magnification using a FViewII digital camera (Olympus Soft Imaging System, Tokyo, Japan) linked to the inverted microscope (Olympus IX81, Japan) equipped with a disk-spinning unit (DSU, Olympus, Japan). Epifluorescence micrographs of stained *M. rubrum* cells were taken at 1,000X magnification using a digital camera coupled to the Olympus BX51 microscope equipped with differential interference contrast. Twenty cells were examined for each sample. 3D images were generated using IMARIS software program (Bitplane, Zürich, Switzerland) to assess the number and volume of chloroplasts of *M. rubrum*.

### Experiment 2: Refeeding of Starved *Mesodinium rubrum*

The second experiment was designed to monitor the changes in the number, size and position of prey nuclei upon refeeding and subsequent prey starvation. After taking subsamples on Day 29, the three cultures from the first experiment were poured together in a 750-ml tissue culture flask and then distributed to three 750-ml tissue flasks. *T. amphioxeia* and new f/2 medium was added to each of the three flasks to give predator:prey ratios of 1:2.5, 1:5, and 1:10 and then a 150-ml subsample of each flask was transferred to each of three 270-ml tissue culture flasks. The triplicate flasks for each treatment were maintained as in Experiment 1, with subsamples for estimating cell abundance (2.3 ml) withdrawn on 10 occasions during the experiment (Day 0, 0.5, 1, 4, 6, 8, 10, 13, 16, and 19) (**Figure 1**). Samples for measuring the size of prey nuclei inside of *M. rubrum* (5–10 ml) were taken on Day 0.5 to Day 19, with Day 29 data from Experiment 1 used as Day 0 data for Experiment 2. All nine flasks were supplied with fresh f/2 medium after removal of half of the ‘old’ medium on Day 10 for the same reason as above.

### Statistical Analyses

Relationships of photosynthesis with prevalence of the centered prey nucleus (CPN; see below) and number of chloroplasts were analyzed and plotted using non-linear regression analysis (Sigma Plot v. 10.0). Data reported in the text as means are given ± standard error of the mean (SE). Error bars provided in figures also represent SE.

## Results

### Experiment 1: Starvation of Well-Fed *Mesodinium rubrum*

#### Nuclei of Prey and Well-Fed *Mesodinium rubrum*

**FIGURE 2:**
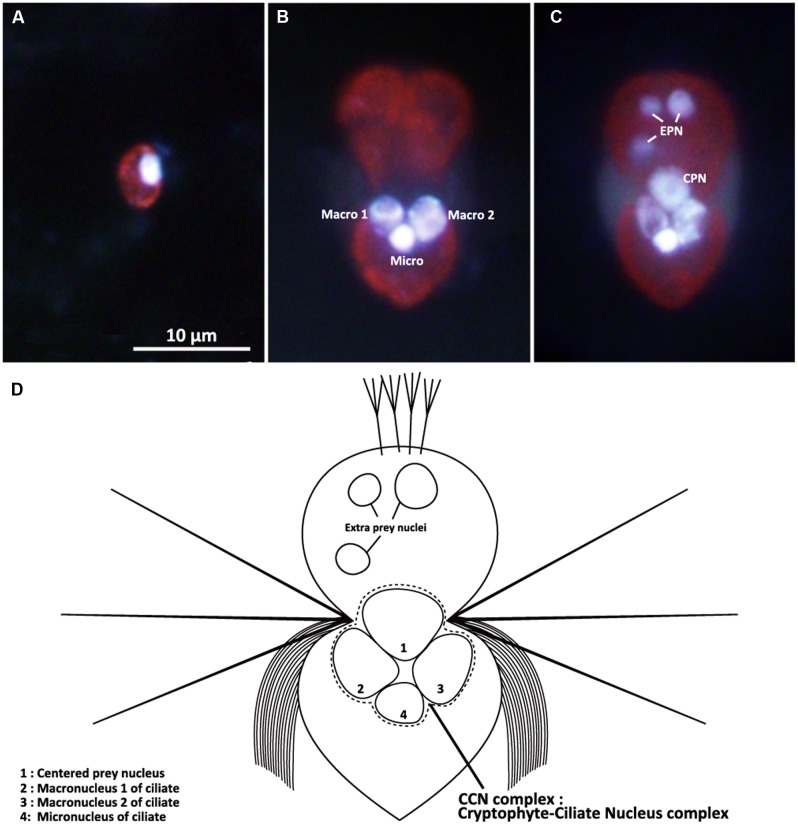
**Epifluorescence micrographs of the cryptophyte *Teleaulax amphioxeia*** **(A)** and the ciliate *Mesodinium rubrum* **(B,C)** stained with both Hoechst 3325 and CellMask Green in combination. **(A)** *T. amphioxeia* showing the brightly stained cryptophyte nucleus. **(B)** Starved *M. rubrum* containing two ciliate macronuclei and one ciliate micronucleus. **(C)** Well-fed *M. rubrum* containing two types of prey nuclei (EPN and CPN) as well as the three nuclei. **(D)** Schematic drawing of the well-fed *M. rubrum*. Macro, ciliate macronucleus; Micro, ciliate micronucleus; EPN, extra prey nucleus; CPN, centered prey nucleus. Scale bar in **(A)** is 10 μm and applies to **(B,C)**.

Well-fed *Mesodinium rubrum* cells (Day 0 to Day 11) contain two ciliate macronuclei and one ciliate micronucleus, all of which were closely positioned at the center of the cell (**Figure 2B**). The two macronuclei (diameter 3.54 μm ± 0.07 μm; **Table 1**) were placed close to each other in the middle of the cell, while the micronucleus (diameter 2.63 μm ± 0.09 μm) was located just posterior to the two macronuclei. Well-fed cells of *M. rubrum* contained additional nuclei of cryptophyte origin. A solitary cryptophyte nucleus (diameter 4.35 μm ± 0.04 μm; **Table 1**) was located in the center of the cell in close association with the ciliate nuclei and always anterior to the two ciliate macronuclei (**Figure 2C**). This arrangement is subsequently referred to as the cryptophyte-ciliate nuclear complex (CCN complex; **Figure 2D**). We also observed smaller cryptophyte nuclei (diameter 2.53 μm ± 0.04 μm), typically located at the periphery of the ciliate and usually in the anterior part of the cell (**Figure 2C**). These nuclei were similar in size to the nucleus (diameter 2.08 μm ± 0.04 μm, **Table 1**) of the prey, *Teleaulax amphioxeia*, (**Figures 2A,C**). We will subsequently refer to the former and latter type of ingested prey nuclei as “CPN” and “extra prey nucleus” (EPN), respectively (**Figure 2C**).

**Table 1:** Diameter of *Teleaulax amphioxeia* nuclei, *Mesodinium rubrum* nuclei, and ingested prey nuclei (EPNs and CPNs) during Experiments 1 and 2.

Species	Type of nucleus		Sample ID	Nuclear diameter^1^ ±*SE* (μm)	Range (μm)	Total # nuclei examined	Total # cells observed
*Teleaulax amphioxeia*	Nucleus		Experiment 1, Day 0	2.08 ± 0.04	1.8 – 2.5	27	27
*Mesodinium rubrum*	Ciliate micronucleus		Experiment 1, Day 0	2.63 ± 0.09	2.3 – 3.8	21	21
	Ciliate macronuclei		Experiment 1, Day 0	3.54 ± 0.07	2.9 – 4.5	42	21
	EPN	Well-fed cells	Experiment 1, Day 0 – Day 8	2.35 ± 0.04	1.3 – 3.9	181	240
		Starved/refeed cells	Experiment 2, Day 0.5 – Day 13	2.17 ± 0.01	1.2 – 4.6	1959	1260
	CPN	Well-fed cells	Experiment 1, Day 0 – Day 11	4.35 ± 0.04	2.5 – 7.7	255	300
		Starved/refeed cells	Experiment 2, Day 0	6.83 + 0.26	5.8 – 8.0	8	60
			Experiment 2, Day 0.5 – Day 4	3.80 + 0.07	1.8 – 8.4	293	540
			Experiment 2, Day 19	5.75 + 0.12	3.9 – 7.6	42	180


*Mean values are for nuclear diameters pooled for specified sample times. ^1^Nuclear diameter estimated as the mean of length and width.*

After staining with a combination of Hoechst 3325 and CellMask Green, the ciliate micronucleus always emitted stronger fluorescence than the two macronuclei (**Figures 2B,C**). EPNs were typically spherical (**Figure 2C**), while CPNs varied from spherical to irregular in shape (**Figure 2C**). EPNs were never clustered close together, and none of 181 EPNs examined during the experiment showed evidence of fusing with another EPN or a CPN. Dividing *M. rubrum* cells were common in stained preparations, but none of 388 CPNs examined for the experiment appeared to be undergoing nuclear division. The location, size, and shape of CPNs present in dividing *M. rubrum* cells (**Figure 9**) were indistinguishable from that of CPNs occurring in non-dividing cells.

#### Cell Divisions

**FIGURE 3:**
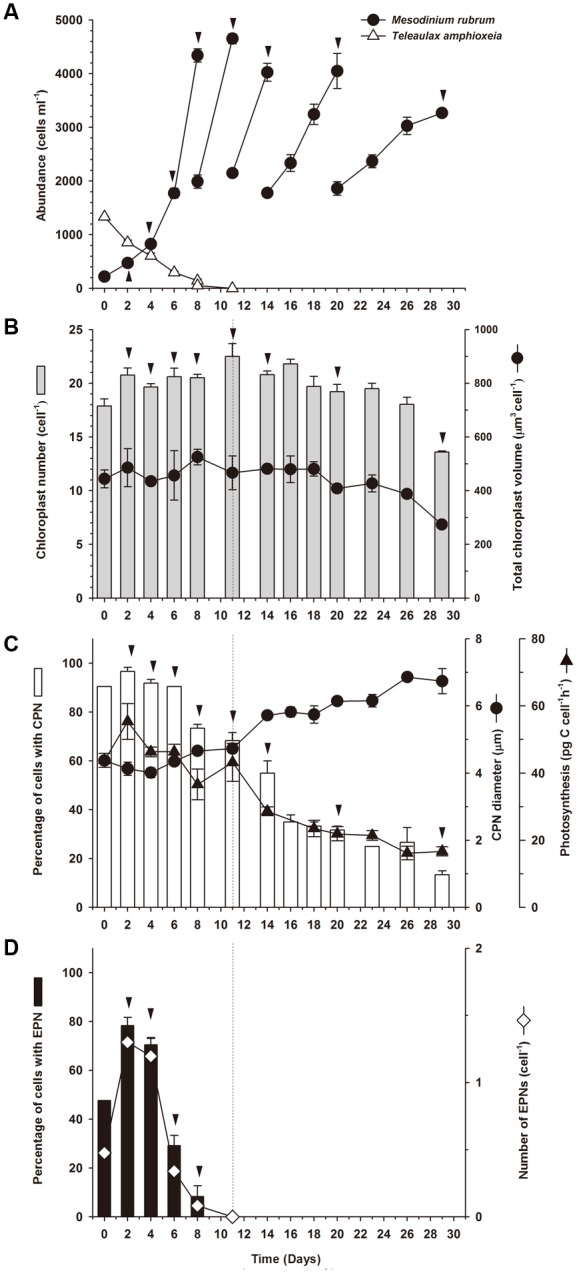
**Experiment 1.** Starvation of well-fed *Mesodinium rubrum*. **(A)** Abundance of *M. rubrum* and *T. amphioxeia* as a function of incubation time. **(B)** Chloroplast number and volume (cell^-1^) as function of incubation time for *M. rubrum*. **(C)** Percentage of *M. rubrum* cells with a centered prey nucleus (CPN), CPN diameter, and photosynthetic rate for *M. rubrum* as a function of incubation time. **(D)** Percentage of *M. rubrum* cells with one or more extra prey nuclei (EPNs) and number of EPNs cell^-1^ over incubation time. Data for cell abundance and photosynthesis represent mean ± SE for triplicate flasks (*n* = 3). Data for other parameters represent mean ± SE for triplicate flasks (*n* = 3), except for Day 0, when cells were examined from a single sample taken prior to distribution of stock culture to experimental flasks. For Day 0, *n* = 18 for chloroplasts number cell^-1^ and chloroplasts volume cell^-1^, *n* = 21 for CPN prevalence, EPN prevalence, CPN number cell^-1^, and EPN number cell^-1^, and *n* = 19 for CPN diameter. Dashed vertical lines in **(B–D)** denote the point at which prey were depleted. Arrowheads indicate a doubling in cell number relative to prior values.

The culture of *Mesodinium rubrum* that was mixed with *Teleaulax amphioxeia* at an initial ratio of 1:5 had almost depleted the cryptophyte prey at Day 8, and the prey were depleted below detection limit (<1 cell ml^-1^) by Day 11 (**Figure 3A**). During the first 8 days of the incubation, *M. rubrum* divided every second day (μ = 0.37 ± 0.01 d^-1^). After that period, growth declined. From Day 8 to Day 14, *M. rubrum* divided every third day (μ = 0.25 ± 0.02 d^-1^), and from Day 14 to end of the experiment cells stopped dividing (μ = 0.10 ± 0.02 d^-1^). A total of eight cell divisions were observed, four of which occurred in the period without available prey.

#### Number and Volume of Chloroplasts

Even though *M. rubrum* divided several times during the experiment, the number of chloroplasts cell^-1^ remained relatively constant at 20 ± 0.39 cell^-1^ (*n* = 12) for 26 days, despite the fact that prey cells were depleted after Day 8 (**Figure 3B**). After Day 26, the number of chloroplasts decreased rapidly to reach 14 ± 0.10 cell^-1^ at the end of the experiment. Chloroplast volume cell^-1^ remained more or less steady from Day 0 to Day 18 (444 ± 33 μm^3^ cell^-1^; *n* = 9), thereafter gradually decreasing to a mean of 274 ± 8 μm^3^ cell^-1^ on the last day of the experiment.

#### Changes in Prevalence and Linear Dimensions of Sequestered Prey Nuclei Inside *M. rubrum* Cells

More than 90% of the well-fed *M. rubrum* cells possessed a CPN at the initiation of the experiment (**Figure 3C**) and a similar percentage was observed for the following 6 days (92.4 ± 0.2%; *n* = 4). The proportion of ciliates having a CPN decreased to ∼70% from Day 8 to 11 as prey cells were depleted, in which time *M. rubrum* underwent two additional cell divisions. Subsequently, the proportion of cells with a CPN gradually declined to ∼13.3% on Day 29.

Centered prey nucleus diameter was relatively stable over the first 11 days of the experiment (**Figure 3C**), showing a minimum value of 4.01 ± 0.15 μm (*n* = 3) for samples taken on Day 4 and a maximum of 4.73 ± 0.14 μm on Day 11 (*n* = 3). During starvation (Day 11–29), however, the size of the CPN gradually increased to 6.8 ± 0.1 μm (*n* = 3) for samples taken on Day 26 (**Figure 3C**), in direct contrast to the change in prevalence of cells with a CPN. The largest CPN observed during the experiment was encountered in samples taken at Day 26 and measured 8.9 μm in diameter.

Extra prey nucleus were only observed in *M. rubrum* cells when prey cells were present (Day 0–8; **Figure 3D**). The percentage of ciliates with EPN was ∼75% at Day 2 and 4, and on average a little more than 1 EPN was found per cell (1.24 ± 0.05, *n* = 2, the number relative to all observed cells). Both values dropped rapidly after Day 4.

#### Inorganic Carbon Uptake

**FIGURE 4:**
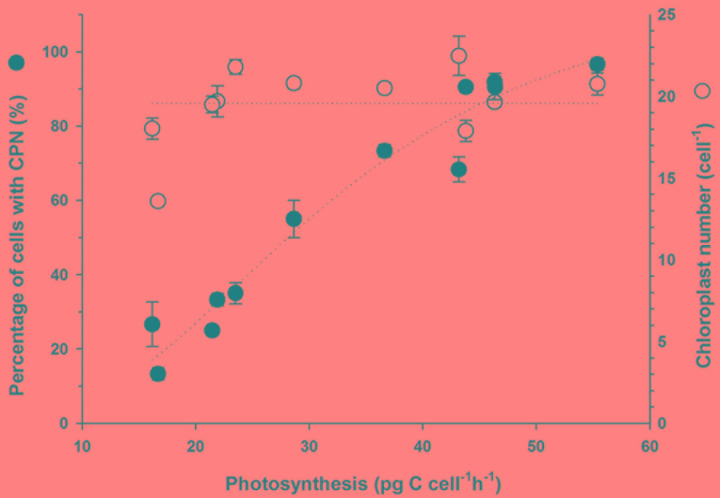
**Prevalence of a centered prey nucleus (CPN) and number of chloroplasts cell^-1^ for *Mesodinium rubrum* in Experiment 1 plotted as a function of photosynthetic rate**.

*Mesodinium rubrum* maintained an inorganic carbon uptake of 45.2 ± 2.5 pg C cell^-1^ h^-1^ (*n* = 6) until Day 11 (**Figure 3C**). Subsequently, photosynthetic activity steadily decreased in conjunction with prey depletion, reaching a value of 16 ± 1.4 pg C cell^-1^ h^-1^ at Day 29. Over the course of the experiment, inorganic carbon uptake displayed a direct relationship to the prevalence of cells with a CPN (*r*^2^ = 0.955, *p* < 0.0001; **Figure 4**). No relationship was observed between inorganic carbon uptake and the number of chloroplasts per cell (*r*^2^ = 0.0, *p* = 1; **Figure 4**).

### Experiment 2: Refeeding of Starved *Mesodinium rubrum*

#### Cell Divisions

**FIGURE 5:**
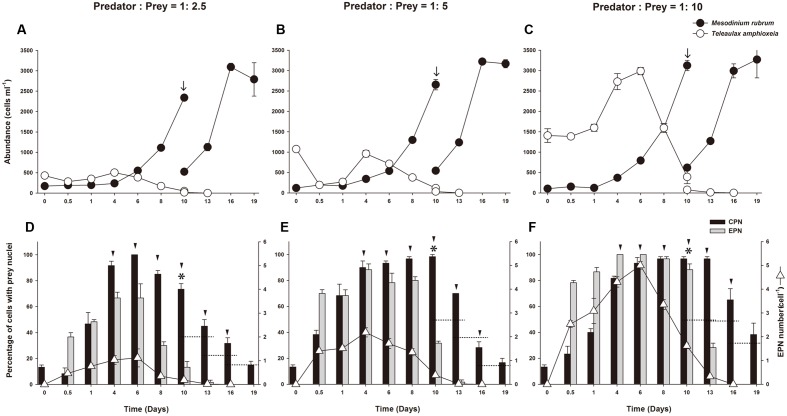
**Experiment 2.** Refeeding of starved *Mesodinium rubrum* **(A–C)**. Abundance of *M. rubrum* and *T. amphioxeia* in low, medium, and high prey treatments, respectively, plotted as a function of incubation time. **(D–F)** Percentage of *M. rubrum* cells with ingested prey nuclei in low, medium, and high prey treatments, respectively, plotted as a function of incubation time. Plots show mean ± SE for triplicate flasks (*n* = 3), with 20 cells examined sample^-1^. EPN, extra prey nucleus; CPN, centered prey nucleus; arrows indicate dilution of experimental cultures; arrowheads indicate a doubling in cell number relative to prior values; stars indicate that prey was present at less than 1% of predator abundance; dotted horizontal lines represent half of the former ratio value.

Predator:prey ratio calculated from mean concentration of the predator during the first 48 h of incubation and initial prey density, was 1:2.5, 1:5, and 1:10 for the different treatments. On Day 10, prey concentrations were less than 1% of initial concentration in all treatments. On Day 13, the prey were depleted below detection limit (<1 cell ml^-1^) in the 1:2.5 and 1:5 treatments, and on Day 16 in the 1:10 treatment (**Figures 5A–C**). *M. rubrum* abundance increased rapidly 4 days after refeeding with prey, showing a doubling in cell concentration every second day until Day 10. All cultures were diluted with fresh f/2 medium to a new initial concentration of 500 cells ml^-1^ on Day 10, resulting in two additional cell divisions every third day, after which growth stopped.

#### Changes in Prevalence of Ingested Prey Nuclei in *M. rubrum* at Different Prey Concentrations

Immediately after the addition of prey, EPNs were observed in *M. rubrum* cells in all treatments. Likewise, when prey was depleted, EPNs were no longer observed (**Figures 5D–F**). Changes in the prevalence and number of EPNs were directly related to prey concentration. The highest percentage of cells having EPNs was observed on Day 4 or Day 6 in the three treatments. At low prey concentration (predator to prey ratio of 1:2.5), 66.6% of *M. rubrum* cells had a mean of 1.12 ± 0.38 (*n* = 3) EPNs cell^-1^ on Day 6 (**Figure 5D**). At moderate prey concentration (predator to prey ratio of 1:5), about 88.3% of *M. rubrum* cells had a mean of 2.18 ± 0.19 (*n* = 3) EPNs cell^-1^ on Day 4 (**Figure 5E**), while at the high initial prey concentration (predator to prey ratio of 1:10), 100% of *M. rubrum* cells retained a mean of 5 ± 0.16 (*n* = 3) EPNs cell^-1^ on Day 6 (**Figure 5F**). The largest number of EPNs retained by a single *M. rubrum* cell (11) was observed on Day 1 in the high prey treatment. The ‘n’ refers to triplicate samples and EPNs were scored in at least 20 cells in each sample.

**FIGURE 6:**
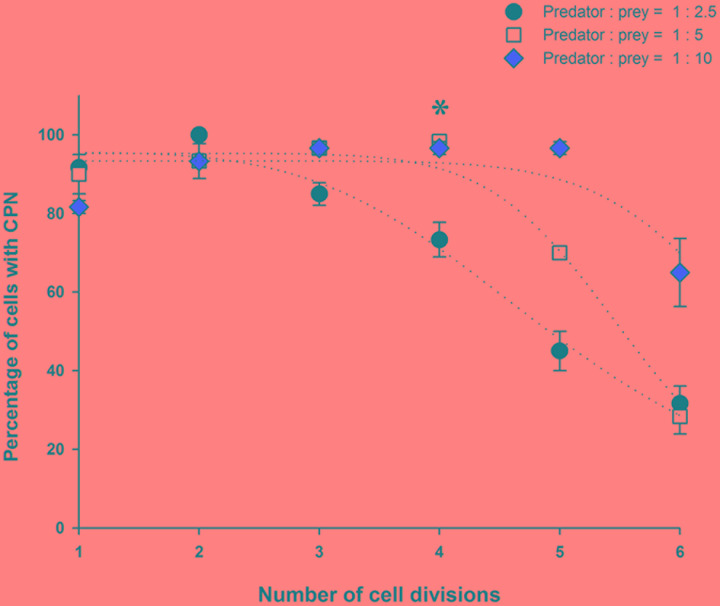
**Prevalence of a centered prey nucleus as a function of the number of cell divisions for low, medium, and high prey treatments of Experiment 2**.

At the start of the experiment, less than 10% of *M. rubrum* cells had a CPN, but the prevalence of cells with a CPN increased after refeeding, exceeding 80% on Day 4 in all treatments (**Figures 5D–F**). Changes in the occurrence of cells with a CPN, however, showed different patterns across the treatments of prey concentration. At the lowest prey density, the prevalence of a CPN declined after Day 6, as cells underwent division (**Figure 5D**), while at moderate prey density, prevalence of a CPN remained above 90% from Day 4 to 10 as cells divided 4 times and then decreased (**Figure 5E**). At the high prey concentration, prevalence of a CPN was maintained above 80% while cells divided five times (Day 4 to Day 13) and then decreased (**Figure 5F**). Changes in the prevalence of CPNs relative to the number of cell divisions were dependent on initial prey density. The percent cells with CPNs declined after three cell divisions in the low prey treatment after 4 cell divisions in the medium prey treatment, and after five cell divisions in the high prey treatment (**Figure 6**).

**FIGURE 7:**
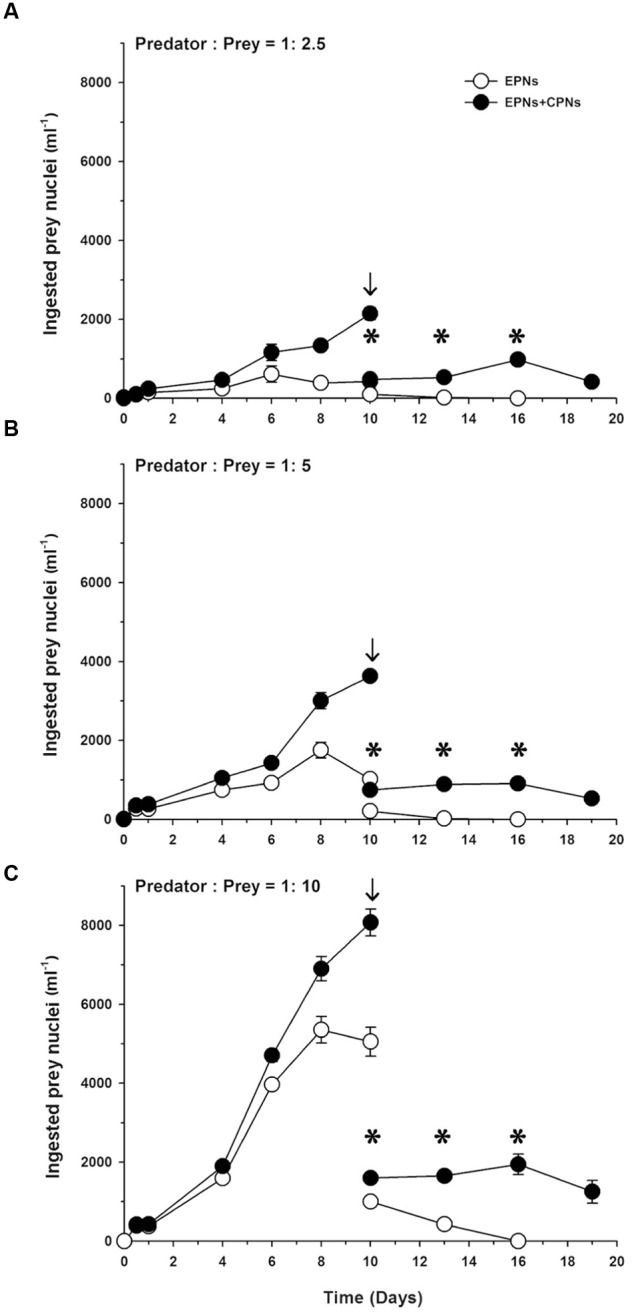
**Abundances of ingested prey nuclei (nuclei ml^-1^) for** **(A)** low, **(B)** medium, and **(C)** high prey treatments of Experiment 2 as a function of incubation time. Mean ± SE for nuclear abundance calculated as mean abundance. *M. rubrum* abundance at each sample time multiplied by number of ingested prey nuclei cell^-1^ for the triplicate flasks (*n* = 3). EPNs, extra prey nuclei; CPNs, center prey nuclei; arrows indicate dilution of cultures; stars indicate relatively constant values for (EPN + CPN) ml^-1^ following dilution of cultures.

Extra prey nucleus abundance (i.e., number ml^-1^) increased to a peak on Day 6 in the low prey treatment and on Day 8 in the medium and high prey treatments, before declining to undetectable levels as prey was depleted (**Figure 7**). Abundance of total ingested prey nuclei (EPNs + CPNs) increased to a peak on Day 10 in all three treatments, but remained relatively stable following dilution of the cultures and depletion of prey. After dilution of the high prey treatment on Day 10, EPN abundance was about half that of total ingested nuclei (**Figure 7C**). Over the following 6 days, EPN abundance in that treatment dropped to zero without influencing abundance of total ingested nuclei.

#### Changes in Size of Sequestered Prey Nuclei in *M. rubrum* at Different Prey Concentrations

**FIGURE 8:**
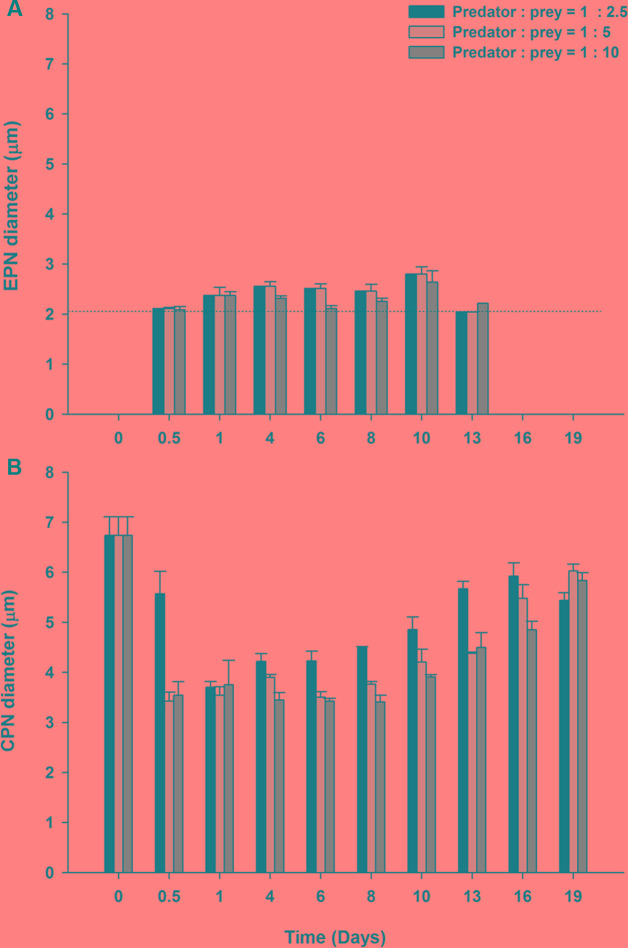
**Mean nuclear diameter ± SE for triplicate flasks (*n* = 3) during Experiment 2.** **(A)** Extra prey nuclei (EPNs) for low, medium, and high prey treatments. **(B)** Centered prey nuclei (CPNs) for low, medium, and high prey treatments. Dotted line in **(A)** denotes the nuclear diameter for the prey, *Teleaulax amphioxeia*, (mean 2.08 μm).

**FIGURE 9:**
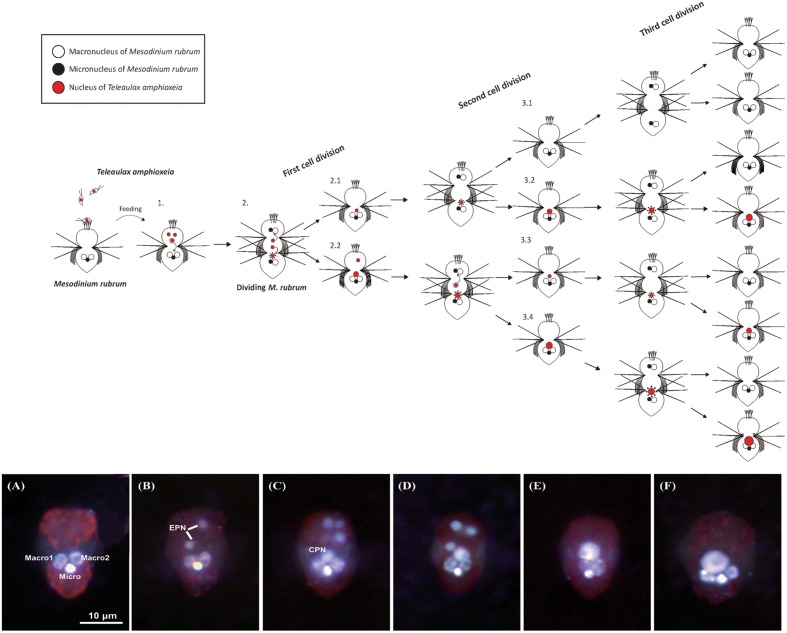
**Schematic diagram showing the ideal dynamics of acquired prey nuclei following cell division in the ciliate *Mesodinium rubrum* and epifluorescence micrographs of *M. rubrum* cells from the high prey treatment of Experiment 2 after staining with Hoechst 33258 and CellMask Green.** (1) *M. rubrum* feeds on three cryptophyte *Teleaulax amphioxeia* cells as prey, and obtains the nuclei of each prey (EPN). One of the EPN moves toward the center of the ciliate (or anterior to the three ciliate nuclei) and becomes enlarged (CPN). The other two EPN transfer to daughter cells through cell division, respectively. After first cell division, one daughter cell (2.1) has a CPN derived from one of the EPN. This daughter cell is divided into new daughter cells having a CPN (3.1) or not (3.2) by a second cell division. Meanwhile, the other daughter cell (2.2) has a CPN with one EPN. In case of this daughter cell, it is divided into new daughter cells through a second cell division; two daughter cells (3.3 and 3.4) have a CPN, respectively. Each CPN moves in the same way as before following a third cell division. As a result, only three cells among the eight daughter cells have a CPN after the third cell division. The series of epifluorescence images show temporal differences in the arrangement, number, and size of ingested prey nuclei after feeding starved *M. rubrum* with *T. amphioxeia.* **(A)** Day 0: 18 days starved *M. rubrum* derived from Day 29 of Experiment 1. **(B)** Day 1, **(C)** Day 4, **(D)** Day 8, **(E)** Day 13, and **(F)** Day 19 after feeding. Macro, macronucleus; Micro, micronucleus; EPN, extra prey nucleus; CPN, centered prey nucleus. Scale bar in **(A)** is 10 μm and applies to all images.

Extra prey nucleus observed in all three treatments were relatively constant in size (**Figure 8A**), with a mean diameter of 2.17 ± 0.01 μm for measurements pooled across treatment and time (**Table 1**). By contrast, CPNs were initially large in size (**Figure 8B**), having a mean diameter of 6.83 ± 0.26 μm on Day 0 (**Table 1**). Treatment means showed decreased CPN size from Day 0.5 to Day 4 as prey were ingested and new CPNs formed, with CPN diameter for data pooled across treatments for those days averaging 3.08 ± 0.07 μm. Like in Experiment 1, the size of CPNs increased over time after prey were depleted (**Figures 8B**, **9**), with data pooled across treatments for Day 19 averaging 5.75 ± 0.12 μm. CPN size at the end of the experiment was similar across treatment, but pronounced enlargement occurred sooner (Day 6–8) in the lowest prey treatment compared to in the medium and high prey treatments (Day 10–13).

Frequency distributions for CPN diameter showed distinct size classes of ‘old’ and ‘new’ CPNs on Day 0 to Day 4. *M. rubrum* on Day 0 were derived from pooled culture remaining on Day 29 of Experiment 1 and thus contained ‘old’ CPNs that fell into the 5.5–6.4 μm to 7.5–8.4 μm size classes (**Supplementary Figure S1**). On Day 3.5, smaller, presumably ‘new’ CPNs were observed, 94% of which fell into the 1.5–2.4 μm and 2.5–3.4 μm size classes and had a mean diameter of 2.58 ± 0.06 μm (*n* = 29). Given the mean diameter of EPNs on Day 0.5 (2.06 μm, **Table 1**), the volume of ‘new’ CPNs (8.9 μm^3^) was about twice that of EPNs (4.5 μm^3^) present in *M. rubrum* at the same time. Over the following 3.5 days, the frequency distribution for ‘new’ CPNs shifted toward larger size classes, with the peak occurring in the 2.5–3.4 μm size class on Day 1 and in the 3.5–4.4 μm size class on Day 4.

A total of 1959 ENPs were examined for the experiment, none of which appeared to be in the process of fusing with another EPN or a CPN. While dividing *M. rubrum* were common in Experiment 2, none of the 335 CPNs examined for Day 0.5 to Day 19 appeared to be undergoing division.

## Discussion

### Presence of Extra Prey Nuclei (ENPs) and the Centered Prey Nucleus

The ciliate *Mesodinium rubrum* has for long been known to harbor prey cytoplasm and cell organelles, including chloroplasts, mitochondria, and nuclei through feeding on cryptophytes (Taylor et al., 1969, 1971; Hibberd, 1977; Oakley and Taylor, 1978). Initially, it was believed that this represented a reduced permanent “symbiont,” but recent molecular studies have indicated that this is not true. There is now clear evidence that the cryptophyte organelles and cytoplasm are identical to the prey that the ciliate is fed (e.g., Johnson et al., 2007) and that the chloroplasts from one cryptophyte species can be exchanged with chloroplasts from another species (Hansen et al., 2012). Previous publications have used transmission electron microscopy and fluorescence microscopy to document the presence of small (1.9–4 μm in diameter) or large (4.5–10 μm in diameter) prey nuclei (Hibberd, 1977; Oakley and Taylor, 1978; Johnson et al., 2007; Kim et al., 2016; Nam et al., 2016). Those reports are limited to observations of only one type of prey nucleus inside individual *M. rubrum* cells and provide little information about how *M. rubrum* retains different sizes of prey nuclei. Kim et al. (2016), however, suggested that small prey nuclei fuse to form a large prey nucleus. Similarly, Nam et al. (2016) reported that small nuclei sequestered from prey were enclosed in a single membrane and suggested that small nuclei moved to the center of the *M. rubrum* cell and fused to form a single large prey nucleus.

Our study documents that along with two ciliate macronuclei and one ciliate micronucleus, well-fed *M. rubrum* cells can simultaneously have multiple small, peripherally positioned prey nuclei that we call extra prey nuclei (EPNs), and a single large, centrally positioned prey nucleus (CPN). EPNs were slightly larger (mean diameter 2.53 μm) than the mean size of the prey, *Teleaulax amphioxeia*, nucleus (mean diameter 2.08 μm). The CPN was always located at the center of the cell, as also shown for the large prey nucleus reported in previous studies (Hibberd, 1977; Hansen and Fenchel, 2006; Kim et al., 2016; Nam et al., 2016), and was generally much larger than the EPNs. The CPN, however, showed considerable variation in size during our experiments, depending on prey concentration and incubation time. The CPN size was relatively stable in well-fed *M. rubrum* supplied with plentiful prey (mean diameter 4.35 μm) but increased dramatically during starvation, averaging 6.8 μm in diameter 18 days after depletion of prey.

#### Formation and Enlargement of the CPN

Eighteen days after depletion of prey, ∼85% of the *M. rubru*m in our study lacked a CPN and none possessed EPNs, but both types of nuclei were reacquired by a majority of the cells upon refeeding. EPNs appeared in most cells within 12 h and increased in number over time, with maximum mean number cell^-1^ ranging from one to five depending on the amount of prey provided. Up to 11 EPNs were observed in individual *M. rubrum*. EPNs occupied a peripheral position in the cell, whereas recently sequestered ‘new’ CPNs were located at the center of the cell had a mean diameter (2.58 μm), similar to that of recently acquired EPNs (2.06 μm) present in cells at the same time. Four days post feeding, 80 – 90%, depending on prey concentration, of *M. rubrum* cells had a CPN, with mean diameter of 3.80 μm, larger than an EPN, but smaller than the CPN of well-fed cells (mean 4.35 μm). Calculations based on mean diameter indicate that the volume of ‘new’ CPNs is about twice that of recently acquired EPNs, raising the possibility that ‘new’ CPNs arise from fusion of two EPNs. However, fusing of multiple EPNs to form a ‘new’ CPN seems unlikely, since none of the more than 2100 EPNs observed in our experiments were closely clustered or appeared to be fusing with another EPN. It seems more probable that ‘new’ CPNs form by the relocation of a single EPN to the center of the cells accompanied by and/or followed by an increase in size that does not result from fusion with another EPN.

One may wonder how the CPN of *M. rubrum* increases in size over time. As mentioned in the introduction, enlargement of the centrally positioned prey nucleus was reported by Johnson et al. (2007), Kim et al. (2016), and Nam et al. (2016) and suggested in the latter two papers to result from the fusion of smaller ingested prey nuclei. Data from our starvation/refeeding study (Experiment 2), however, do not support that hypothesis, for several reasons. First, of the more than 2100 EPNs and over 600 CPNs examined during our experiments, fusions of an EPN with a CPN was never observed. If enlargement of the ‘new’ CPN were to be a slow process, fusion events might occur infrequently and thus not be observed in our samples. Enlargement of ‘new’ CPNs in our starvation/refeeding study, however, appeared to be a rather rapid process, as indicated by the observed upward shift in size classes of ‘new’ CPNs from Day 0.5 to Day 1 and Day 4. Second, during both of our experiments, CPNs showed a dramatic increase after prey had been depleted and continued to enlarge even after the number of EPNs cell^-1^ had dropped to undetectable levels. Enlargement of CPNs when prey cells were not available to be ingested and when EPNs were not present in *M. rubrum* cells, indicates that CPNs can enlarge without fusion with EPNs. Third, once prey were reduced to very low or undetectable levels, the number of total ingested prey nuclei (EPNs + CPNs) ml^-1^ remained stable as the number of EPNs ml^-1^ decreased. Were the disappearance of EPNs to result from fusion with CPNs, then the total number of ingested prey nuclei ml^-1^ would be expected to decrease. Fourth, if enlargement of CPNs were to result from fusion with EPNs, then it would be reasonable to expect the size of the CPNs and the rate of CPN enlargement to depend on the number of EPNs that are retained. In our starvation/refeeding study, however, the size CPNs was similar in all treatments even though the maximum mean number of EPNs cell^-1^ showed a fivefold difference. In addition, enlargement of CPNs progressed faster in our lowest prey treatment where maximum mean number of EPNs cell^-1^ was one, than in our moderate and high prey treatment where maximum mean number of EPNs cell^-1^ was two and five, respectively (**Figure 8B**).

Our results support the alternative hypothesis that CPNs are formed by the relocation of a single EPN from the periphery to the center of the *M. rubrum* cell where it becomes part of the ciliate-cryptophyte nuclear complex and increases in size without fusing with EPNs. Under that scenario, EPNs remaining at the periphery of cells after formation of a ‘new’ CPN might be digested, or might be distributed to daughter cells during division of *M. rubrum* where they would be available to form a ‘new’ CPN. The latter possibility assumes that CPNs do not divide along with the ciliate, as our data suggest (see below). If EPNs are distributed to daughter cells to form ‘new’ CPN, then the mean number of EPNs cell^-1^ would influence the number of cell divisions that could occur without a decline in the frequency of *M. rubrum* with a CPN. That seems to be the case in our starvation/refeeding experiment, as *M. rubrum* in the low, medium, and high prey treatments had a maximum mean number EPNs cell^-1^ of 1, 2, and 5 divided 3, 4, and 5 times, respectively, before showing a drop in CPN frequency.

#### Lack of Division and Disappearance of the CPN

Our results imply that the CPN of *M. rubrum* does not divide. Not only did we fail to observe indications of division in any of the more than 600 CPNs examined, CPNs of dividing *M. rubrum* were indistinguishable from the CPNs of non-dividing cells. Also, CPN prevalence decreased with division of host cells as prey were depleted, suggesting dilution of CPN cell^-1^ due to lack of CPN division. As mentioned above, the total number of ingested prey nuclei (EPNs + CPNs) ml^-1^ as prey were depleted remained relatively constant, due to apparent transformation of EPNs into CPNs. Were CPNs to have divided during that time, total ingested prey nuclei ml^-1^ should have increased. Hence, during cell division of *M. rubrum*, the CPN appears to be inherited by only one of the two daughter cells. The lack of the ability of *M. rubrum* to divide the CPN has previously been reported by Johnson et al. (2007). During prey starvation they found a disappearance of the CPN (termed kleptokaryon in their study) in *M. rubrum* over time and could estimate a half time of its disappearance. Hansen and Fenchel (2006) and Kim et al. (2016), asserted that the CPN in *M. rubrum* is able to divide at least once in prey starved cultures, since cells that had undergone one cell division all had a CPN. Based on our observations, however, their results could be explained by the retention of EPNs prior to cell division and distribution of EPN to daughter cells to generate CPNs, rather than by division of the CPNs.

Through the present study, enlargement of the CPN was inferred to result from an increase in size of only one EPN. However, one question still remains to be answered: what causes enlargement of the CPN. Prior studies (Hibberd, 1977; Kim et al., 2016; Nam et al., 2016) have provided ultrastructural images of the enlarged prey nucleus (i.e., CPN) within *M. rubrum*. Here the chromosomes seemed to be untangled or less dense, the nucleolus had a large size and large amounts of nucleoplasm surrounded the expanded nuclear envelope. We also observed similar morphological changes of the CPN under the confocal microscope, with less dense chromosomes and an enlarged nucleolus present in large irregular shaped CPNs (**Supplementary Figure S2**). The chromosome just seemed to be loose or swollen, but the possibility of replication cannot be ruled out. Surprisingly, two enlarged nucleoli were found in one CPN, a phenomenon that also appeared in the study of Johnson et al. (2007). Nevertheless our quantitative data do not indicate that *M. rubrum* can replicate the CPN and the results seem inconsistent with a recent study by Qiu et al. (2016). They observed gene transcripts for prey nucleus replication on field populations of *M. rubrum*. Since their study was carried out on field populations, it is difficult to interpret their data. It is possible that *M. rubrum* population they studied function differently than those isolates from Korea, Denmark, and Antarctica that have been studied in detail in laboratory culture. After all six clades (Clades A–F) of the *M. rubrum*/*M. major* species complex have been described recently (Herfort et al., 2011; Johnson et al., 2016). However, it is also possible that the *M. rubrum* population studied by Qiu et al. (2016) functions similar to the ones studied in detail in the laboratory, but the authors may have simply caught *M. rubrum* cells that recently ingested a diving cryptophyte cell. Future studies will show which of the two interpretations are right.

#### Prey Nucleus Effects on Photosynthetic Ability of *M. rubrum*

*Mesodinium rubrum* is unique among the marine alveolates for its ability to sequester prey nuclei and chloroplasts along with other organelles and show enlargement of the prey nucleus once sequestered. While sequestration of nuclei and chloroplasts along with other prey organelles is well known for a few dinoflagellates species (Dodge, 1971; Farmer and Roberts, 1990; Horiguchi and Pienaar, 1992; Okamoto and Inouye, 2005; Gast et al., 2007; Yamaguchi et al., 2011; Onuma and Horiguchi, 2013, 2015; Kim et al., 2014), enlargement of the sequestered prey nucleus has not been reported in any of studies.

The sequestered prey nucleus has been inferred to allow the host cell to exploit photosynthetic performance of its sequestered prey chloroplasts; e.g., the dinoflagellates *Amylax triacantha*, *Nusuttodinium aeruginosum*, and *N. myriopyrenoides* and the ciliate *M. rubrum* (Johnson and Stoecker, 2005; Johnson et al., 2007; Kim et al., 2014, 2016; Onuma and Horiguchi, 2015). A few molecular and transcriptome studies focusing on the photosynthetic ability of *M. rubrum* in association with the sequestered prey nucleus (Johnson et al., 2007; Lasek-Nesselquist et al., 2015; Kim et al., 2016) have shown expression of nuclear-encoded plastid-targeted algal genes. Subsequently, the retained prey nucleus was suggested to mainly contribute to sustained chloroplasts function (Johnson et al., 2007; Hansen et al., 2012; Lasek-Nesselquist et al., 2015; Kim et al., 2016). It has previously been shown that photosynthetic activity in different *M. rubrum* strains declines in prey starved cultures (Johnson and Stoecker, 2005; Hansen and Fenchel, 2006; Johnson et al., 2007). It has also been shown that the declines in photosynthetic parameters coincided with the loss of CPN (called prey nuclei) from *M. rubrum* cells, implicating a possible functional role for retained prey nuclei (Johnson and Stoecker, 2005; Johnson et al., 2007). Our work confirms that the presence of a CPN substantially affects the photosynthetic performance of the *M. rubrum* chloroplasts. A reduction in prevalence of the CPNs in starved populations of *M. rubrum* led to a significant decline in inorganic carbon uptake while chloroplast number cell^-1^ showed little change. This result might help to explain the formation of the ‘CCN’ complex; the position could facilitate the gene exchange related with nuclear-encoded, chloroplasts targeted genes for stable photosynthesis, between the host nuclei and prey nucleus (Martin et al., 1998; Martin and Herrmann, 1998). Even though our results imply that a CPN is involved with photosynthetic ability of *M. rubrum*, it cannot be ruled out that the ciliate nuclei also participate in the photosynthetic ability of *M. rubrum*. We do not know whether retention of a CPN and chloroplasts derived from prey in *M. rubrum* is an evolutionary step toward establishing permanent chloroplasts, but *M. rubrum* is notable for showing the unique photosynthetic performance from the acquired chloroplasts and nucleus of prey.

Hansen et al. (2012) proved that *M. rubrum* chloroplasts derived from *T. amphioxeia* can be replaced by chloroplasts derived from *T. acuta*. Whether or not the sequestered prey nucleus was simultaneously replaced, however, remains unknown. Addressing the possibility of replacement of the prey nucleus in *M. rubrum* may enhance our understanding of sequestration, enlargement, and function of the prey nucleus inside *M. rubrum*.

#### Model for CPN Maintenance and Increase in Size

Based on the results of our study, we propose the following model to explain the dynamics of acquisition, enlargement, and distribution of prey nuclei to daughter cells in *Mesodinium rubrum*. When *M. rubrum* having only three ciliate nuclei (one micronucleus and two macronuclei positioned at the center of the cell) feeds on the cryptophyte *Teleaulax amphioxeia*, prey nuclei (i.e., EPNs) are acquired at the periphery of the cell. The number of acquired nuclei is equal to the number of prey ingested (**Figure 9**). Subsequently, one of the EPNs relocates to the center of the cell (or anterior to the three ciliate nuclei) to become a CPN, thus forming a CCN complex. The newly formed CPN is small at first, but continuously increases in size over time, without fusing with EPNs that persist in the cell. With division of *M. rubrum*, the enlarged CPN does not divide and is inherited by only one of the daughter cells, with the other daughter cell having the possibility of receiving one or more of the persisting EPNs. If the daughter cell lacking a CPN receives one or more EPNs, then a single EPN relocates to the center of the cell to form a CCN complex and a CPN which enlarges over time. The inherited CPN continues to enlarge until reaching maximum size as seen in starved *M. rubrum*. Once the ‘old’ inherited CPN senesces, it can be replaced by a new CPN if one or more EPNs are present in the peripheral cytoplasm of *M. rubrum* or are acquired through feeding.

## Conclusion

Our study show that the sequestered prey nucleus (CPN) associated with the CCN complex (Cryptophyte-Ciliate Nuclear complex) of *M. rubrum* is derived from a single prey nucleus, enlarges over time without fusing with other ingested prey nuclei (EPNs), does not divide, and is inherited by only one daughter cell when *M. rubrum* divides. Also, EPNs present in *M. rubrum* possessing a CPN can be inherited by and form a CPN in the daughter cell that does not receive the parental CPN. How the EPN-CPN system of *M. rubrum* works when the ciliate is fed a mixture of prey species is unknown. Will *M. rubrum* then contain two CPNs or can one CPN control the chloroplasts from two species? To elucidate this, development of species specific molecular techniques is required. Our study was conducted using *M. rubrum* isolated from Danish waters, while reports of fusion of ingested prey nuclei to form the enlarged prey nucleus (i.e., CPN) of the CCN complex (Kim et al., 2016; Nam et al., 2016) was based on a Korean strain (MR-MAL01) of *Mesodinium* cf. *rubrum*. Since our Danish *M. rubrum* and the Korean *M*. cf. *rubrum* differ at the strain level and may even represent different species, it is possible that the two cultures process ingested prey nuclei in different way. Additional studies using the Korean isolate of *M.* cf. *rubrum* and other isolates of *M. rubrum* from other parts of the world may help to resolve this issue.

## Author Contributions

All authors were involved in the design and planning of the experiments. MK carried out the experiments, collected data, and performed data analysis with help from the co-authors. MK wrote the first draft of the manuscript based on discussions with all the co-authors. All co-authors were involved in revision of the manuscript and all the co-authors approved final manuscript.

## Conflict of Interest Statement

The authors declare that the research was conducted in the absence of any commercial or financial relationships that could be construed as a potential conflict of interest. The reviewer MJ declared a past co-authorship with several of the authors (MK, KD, PJH) to the handling Editor, who ensured that the process met the standards of a fair and objective review.

## Supplementary Material

**FIGURE S1 | Frequency distributions for diameter of centered prey nuclei (CPNs) on** **(A)** Day 0, **(B)** Day 0.5, **(C)** Day 1, and **(D)** Day 4 of Experiment 2. “Old CPNs” on Day 0 are enlarged nuclei remaining in *M. rubrum* cells after 18 days of starvation (Day 29 of Experiment 1). “New CPNs” represent nuclei acquired after addition of prey at the beginning of Experiment 2. In **(B–D)**, CPNs in the three largest size classes were assumed to be “old” CPNs.

**FIGURE S2 | Confocal images showing differences in size and shape of ingested prey nucleus in *M. rubrum*** **(A)** 2 days, **(B–D)** 29 days after feeding on prey. **(A)** Ingested prey nucleus located at the periphery of the ciliate (EPN) similar to the nucleus of the prey species. **(B)** Enlarged prey nucleus located at the center of the cell (CPN) seems to have untangled or less dense chromosomes and enlarged nucleolus. **(C)** The CPN shows morphological change. **(D)** The CPN has two enlarged nucleoli. Scale bar in **(A)** is 5 μm and applies to all images.

## References

[B1] Dodge J. D. (1971). A dinoflagellate with both a mesocaryotic and a eucaryotic nucleus I. Fine structure of the nuclei. *Protoplasma* 73 145–157. 10.1007/BF01275591 5126401

[B2] Farmer M. A. Roberts K. R. (1990). Organelle loss in the endosymbiont of *Gymnodinium acidotum* (Dinophyceae). *Protoplasma* 153 178–185. 10.1007/BF01354002

[B3] Gast R. J. Moran D. M. Dennett M. R. Caron D. A. (2007). Kleptoplasty in an Antarctic dinoflagellate: caught in evolutionary transition? *Environ. Microbiol.* 9 39–45. 10.1111/j.1462-2920.2006.01109.x 17227410

[B4] Guillard R. R. (1975). “Culture of phytoplankton for feeding marine invertebrates,” in *Culture of Marine Invertebrate Animals*, eds Smith W. L. Chanley M. H. (New York, NY: Springer), 29–60. 10.1007/978-1-4615-8714-9_3

[B5] Gustafson D. E. Stoecker D. K. Johnson M. D. Van Heukelem W. F. Sneider K. (2000). Cryptophyte algae are robbed of their organelles by the marine ciliate *Mesodinium rubrum*. *Nature* 405 1049–1052. 10.1038/35016570 10890444

[B6] Hansen P. J. (2002). Effect of high pH on the growth and survival of marine phytoplankton: implications for species succession. *Aquat. Microb. Ecol.* 28 279–288. 10.3354/ame028279

[B7] Hansen P. J. Fenchel T. (2006). The bloom-forming ciliate *Mesodinium rubrum* harbours a single permanent endosymbiont. *Mar. Biol. Res.* 2 169–177. 10.1080/17451000600719577

[B8] Hansen P. J. Moldrup M. Tarangkoon W. Garcia-Cuetos L. Moestrup Ø. (2012). Direct evidence for symbiont sequestration in the marine red tide ciliate *Mesodinium rubrum*. *Aquat. Microb. Ecol.* 66 63–75. 10.3354/ame01559

[B9] Herfort L. Peterson T. D. McCue L. A. Crump B. C. Prahl F. G. Baptista A. M. (2011). *Myrionecta rubra* population genetic diversity and its cryptophyte chloroplast specificity in recurrent red tides in the Columbia River estuary. *Aquat. Microb. Ecol.* 62 85–97. 10.3354/ame01460

[B10] Hibberd D. J. (1977). Observations on the ultrastructure of the cryptomonad endosymbiont of the red-water ciliate *Mesodinium rubrum*. *J. Mar. Biol. Assoc. U.K.* 57 45–61. 10.1017/S0025315400021226

[B11] Horiguchi T. Pienaar R. N. (1992). *Amphidinium latum* Lebour (Dinophyceae), a sand-dwelling dinoflagellate feeding on cryptomonads. *Jpn. J. Phycol.* 40 353–363.

[B12] Johnson M. D. (2011). Acquired phototrophy in ciliates: a review of cellular interactions and structural adaptations. *J. Eukaryot. Microbiol.* 58 185–195. 10.1111/j.1550-7408.2011.00545.x 21518077

[B13] Johnson M. D. Beaudoin D. J. Laza-Martinez A. Dyhrman S. T. Fensin E. Lin S. (2016). The genetic diversity of *Mesodinium* and associated Cryptophytes. *Front. Microbiol.* 7:2017 10.3389/fmicb.2016.02017 PMC5168500 28066344

[B14] Johnson M. D. Oldach D. Delwiche C. F. Stoecker D. K. (2007). Retention of transcriptionally active cryptophyte nuclei by the ciliate *Myrionecta rubra*. *Nature* 445 426–428. 10.1038/nature05496 17251979

[B15] Johnson M. D. Stoecker D. K. (2005). Role of feeding in growth and photophysiology of *Myrionecta rubra*. *Aquat. Microb. Ecol.* 39 303–312. 10.3354/ame039303

[B16] Johnson M. D. Tengs T. Oldach D. Stoecker D. K. (2006). Sequestration, performance, and functional control of cryptophyte plastids in the ciliate *Myrionecta rubra* (Ciliophora). *J. Phycol.* 42 1235–1246. 10.1111/j.1529-8817.2006.00275.x

[B17] Kim G. H. Han J. H. Kim B. Han J. W. Nam S. W. Shin W. (2016). Cryptophyte gene regulation in the kleptoplastidic, karyokleptic ciliate *Mesodinium rubrum*. *Harmful Algae* 52 23–33. 10.1016/j.hal.2015.12.004 28073468

[B18] Kim M. Kim K. Y. Nam S. W. Shin W. Yih W. Park M. G. (2014). The effect of starvation on plastid number and photosynthetic performance in the kleptoplastidic dinoflagellate *Amylax triacantha*. *J. Eukaryot. Microbiol.* 61 354–363. 10.1111/jeu.12115 24734883

[B19] Lasek-Nesselquist E. Wisecaver J. H. Hackett J. D. Johnson M. D. (2015). Insights into transcriptional changes that accompany organelle sequestration from the stolen nucleus of *Mesodinium rubrum*. *BMC Genomics* 16:805 10.1186/s12864-015-2052-9 PMC4609049 26475598

[B20] Lindholm T. (1985). *Mesodinium rubrum*-a unique photosynthetic ciliate. *Adv. Aquat. Microbiol.* 3 1–48.

[B21] Martin W. Herrmann R. G. (1998). Gene transfer from organelles to the nucleus: How much, what happens, and why? *Plant Physiol.* 118 9–17. 10.1104/pp.118.1.9 9733521 PMC1539188

[B22] Martin W. Stoebe B. Goremykin V. Hansmann S. Hasegawa M. Kowallik K. V. (1998). Gene transfer to the nucleus and the evolution of chloroplasts. *Nature* 393 162–165. 10.1038/30234 11560168

[B23] Moeller H. V. Johnson M. D. Falkowski P. G. (2011). Photoacclimation in the phototrophic marine ciliate *Mesodinium rubrum* (Ciliophora). *J. Phycol.* 47 324–332. 10.1111/j.1529-8817.2010.00954.x 27021864

[B24] Myung G. Kim H. S. Park J. S. Park M. G. Yih W. (2011). Population growth and plastid type of *Myrionecta rubra* depend on the kinds of available cryptomonad prey. *Harmful Algae* 10 536–541. 10.1016/j.hal.2011.04.005

[B25] Myung G. Kim H. S. Park J. W. Park J. S. Yih W. (2013). Sequestered plastids in *Mesodinium rubrum* are functionally active up to 80 days of phototrophic growth without cryptomonad prey. *Harmful Algae* 27 82–87. 10.1016/j.hal.2013.05.001

[B26] Nam S. W. Park J. W. Yih W. Park M. G. Shin W. (2016). The fate of cryptophyte cell organelles in the ciliate *Mesodinium* cf. *rubrum* subjected to starvation. *Harmful Algae* 59 19–30. 10.1016/j.hal.2016.09.002 28073503

[B27] Oakley B. R. Taylor F. J. R. (1978). Evidence for a new type of endosymbiotic organization in a population of the ciliate *Mesodinium rubrum* from British Columbia. *BioSystems* 10 361–369. 10.1016/0303-2647(78)90019-9 747751

[B28] Okamoto N. Inouye I. (2005). A secondary symbiosis in progress? *Science* 310 287–287. 16224014 10.1126/science.1116125

[B29] Onuma R. Horiguchi T. (2013). Morphological transition in kleptochloroplasts after ingestion in the dinoflagellates *Amphidinium poecilochroum* and *Gymnodinium aeruginosum* (Dinophyceae). *Protist* 164 622–642. 10.1016/j.protis.2013.06.003 23880436

[B30] Onuma R. Horiguchi T. (2015). Kleptochloroplast enlargement, karyoklepty and the distribution of the cryptomonad nucleus in *Nusuttodinium* (= *Gymnodinium*) *aeruginosum* (Dinophyceae). *Protist* 166 177–195. 10.1016/j.protis.2015.01.004 25771111

[B31] Park J. S. Myung G. Kim H. S. Cho B. C. Yih W. (2007). Growth responses of the marine photosynthetic ciliate *Myrionecta rubra* to different cryptomonad strains. *Aquat. Microb. Ecol.* 48 83–90. 10.3354/ame048083

[B32] Qiu D. Huang L. Lin S. (2016). Cryptophyte farming by symbiotic ciliate host detected in situ. *Proc. Natl. Acad. Sci. U.S.A.* 113 12208–12213. 10.1073/pnas.1612483113 27791006 PMC5087057

[B33] Raho N. Jaén D. Mamán L. Rial P. Marín I. (2014). psbA based molecular analysis of cross-feeding experiments suggests that *Dinophysis acuta* does not harbour permanent plastids. *Harmful Algae* 35 20–28. 10.1016/j.hal.2014.03.003

[B34] Skovgaard A. Hansen P. J. Stoecker D. K. (2000). Physiology of the mixotrophic dinoflagellate *Fragilidium subglobosum*. I. Effects of phagotrophy and irradiance on photosynthesis and carbon content. *Mar. Ecol. Prog. Ser.* 201 129–136. 10.3354/meps201129

[B35] Smith M. Hansen P. J. (2007). Interaction between *Mesodinium rubrum* and its prey: importance of prey concentration, irradiance and pH. *Mar. Ecol. Prog. Ser.* 338 61–70. 10.3354/meps338061

[B36] Taylor F. J. R. Blackbourn D. J. Blackbourn J. (1969). Ultrastructure of the chloroplasts and associated structures within the marine ciliate *Mesodinium rubrum* (Lohmann). *Nature* 224 819–821. 10.1038/224819a0

[B37] Taylor F. J. R. Blackbourn D. J. Blackbourn J. (1971). The red-water ciliate *Mesodinium rubrum* and its “incomplete symbionts”: a review including new ultrastructural observations. *J. Fish. Res. Board Can.* 28 391–407. 10.1139/f71-052

[B38] Yamaguchi H. Nakayama T. Kai A. Inouye I. (2011). Taxonomy and phylogeny of a new kleptoplastidal dinoflagellate, *Gymnodinium myriopyrenoides* sp. nov. (Gymnodiniales, Dinophyceae), and its cryptophyte symbiont. *Protist* 162 650–667. 10.1016/j.protis.2011.01.002 21497133

[B39] Yih W. Kim H. S. Jeong H. J. Myung G. Kim Y. G. (2004). Ingestion of cryptophyte cells by the marine photosynthetic ciliate *Mesodinium rubrum*. *Aquat. Microb. Ecol.* 36 165–170. 10.3354/ame036165

