# Supplementary figures and images for: Dynamics of Sequestered Cryptophyte Nuclei in Mesodinium rubrum during Starvation and Refeeding
Source: Front Microbiol. 2017 Mar 21;8:423. doi: 10.3389/fmicb.2017.00423 (PMC5359308; doi:10.3389/fmicb.2017.00423)

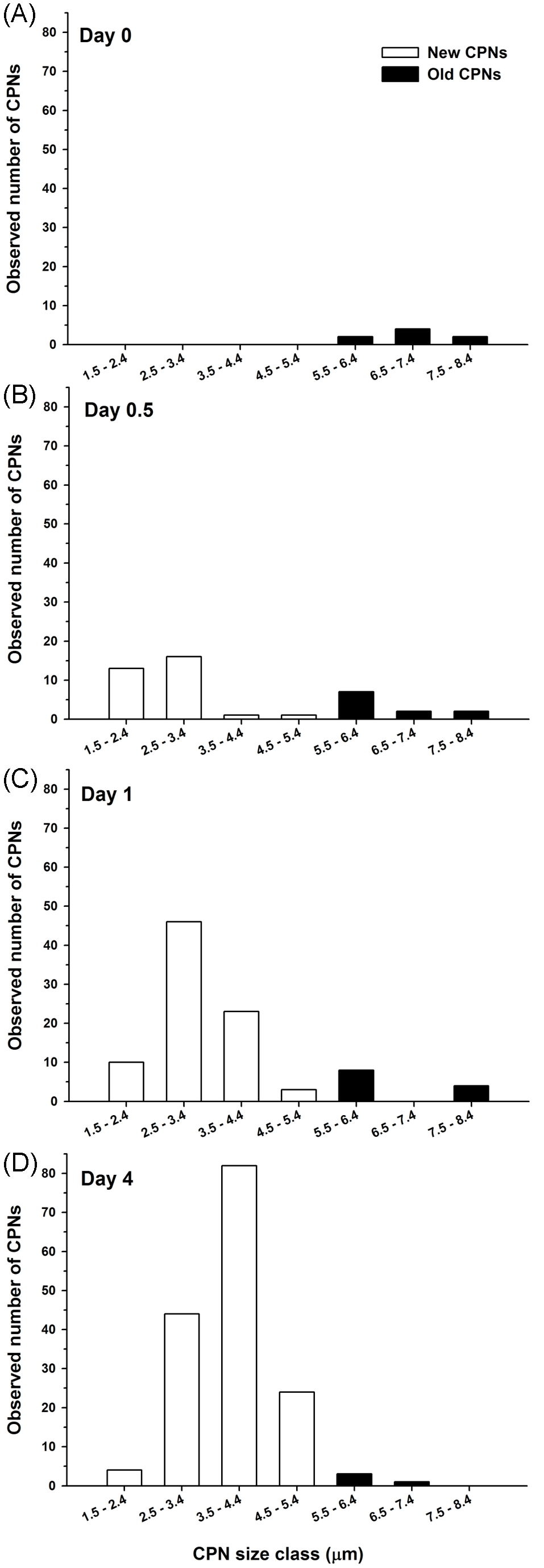

Supplement: Supplementary file 1 [file Image_1.tif]

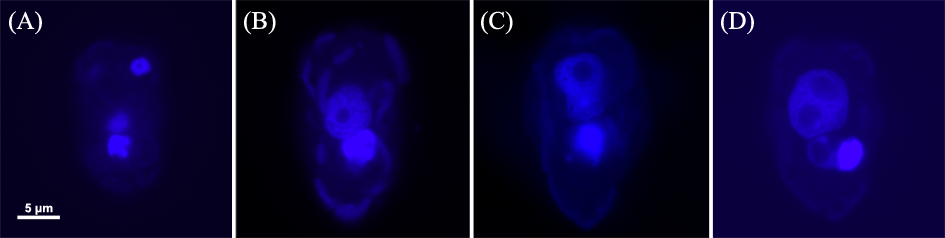

Supplement: Supplementary file 2 [file Image_2.TIF]
